# Cross clinical-experimental-computational qualification of *in silico* drug trials on human cardiac purkinje cells for proarrhythmia risk prediction

**DOI:** 10.3389/ftox.2022.992650

**Published:** 2022-10-05

**Authors:** Cristian Trovato, Marcel Mohr, Friedemann Schmidt, Elisa Passini, Blanca Rodriguez

**Affiliations:** ^1^ Department of Computer Science, University of Oxford, Oxford, United Kingdom; ^2^ Sanofi-Aventis Deutschland GmbH, R&D Preclinical Safety, Frankfurt, Germany

**Keywords:** *in silico* trials, cardiac electrophysiology, computer modelling, drug safety, purkinje fibre, cardiac arrythmia, cardiac action potential, hERG

## Abstract

The preclinical identification of drug-induced cardiotoxicity and its translation into human risk are still major challenges in pharmaceutical drug discovery. The ICH S7B Guideline and Q&A on Clinical and Nonclinical Evaluation of QT/QTc Interval Prolongation and Proarrhythmic Potential promotes human *in silico* drug trials as a novel tool for proarrhythmia risk assessment. To facilitate the use of *in silico* data in regulatory submissions, explanatory control compounds should be tested and documented to demonstrate consistency between predictions and the historic validation data. This study aims to quantify drug-induced electrophysiological effects on *in silico* cardiac human Purkinje cells, to compare them with existing *in vitro* rabbit data, and to assess their accuracy for clinical pro-arrhythmic risk predictions. The effects of 14 reference compounds were quantified in simulations with a population of *in silico* human cardiac Purkinje models. For each drug dose, five electrophysiological biomarkers were quantified at three pacing frequencies, and results compared with available *in vitro* experiments and clinical proarrhythmia reports. Three key results were obtained: 1) *In silico,* repolarization abnormalities in human Purkinje simulations predicted drug-induced arrhythmia for all risky compounds, showing higher predicted accuracy than rabbit experiments; 2) Drug-induced electrophysiological changes observed in human-based simulations showed a high degree of consistency with *in vitro* rabbit recordings at all pacing frequencies, and depolarization velocity and action potential duration were the most consistent biomarkers; 3) discrepancies observed for dofetilide, sotalol and terfenadine are mainly caused by species differences between humans and rabbit. Taken together, this study demonstrates higher accuracy of *in silico* methods compared to *in vitro* animal models for pro-arrhythmic risk prediction, as well as a high degree of consistency with *in vitro* experiments commonly used in safety pharmacology, supporting the potential for industrial and regulatory adoption of *in silico* trials for proarrhythmia prediction.

## 1 Introduction

Preclinical assessment of drug-induced arrhythmia or proarrhythmia is a key requirement for pharmaceutical industries and regulators. This is particularly relevant for compounds showing a positive hERG (human Ether-à-go-go-Related Gene) signal, but also blocking other ionic channels (Gintant et al., 2016). The current ICH S7B/E14 guidelines have prevented new pro-arrhythmic drugs from entering the market, though, they have also led to premature termination of drug development (and potentially of valuable therapeutics) based solely upon either the hERG assay or thorough-QT study results (Lester and Olbertz, 2016). hERG encodes the potassium channel related to the rapidly activating delayed rectifier potassium current (I_Kr_), which - when blocked - leads to prolongation of the QT segment of the ECG, and potentially to arrhythmia. Predicting proarrhythmia is challenging, due to the interplay of different ionic currents underlying the cellular electrical activity, i.e., the action potential (AP), and the complex drug-ionic channels interactions.

*In vitro*, *in vivo*, and *ex-vivo* animal models are widely used for preclinical proarrhythmia assessment, often considering metrics based on drug-induced AP prolongation as a surrogate of QT prolongation. Among these, cardiac Purkinje fibers obtained from dog or rabbit hearts, are one of the most established and ICH S7B-recommended *in vitro* models for preclinical cardiotoxicity screening (European Medical Agency 2006; Roche et al., 2010). However, species differences between animals and humans, limit the accuracy of animal models for clinical risk prediction, in addition to other limitations such as the hefty cost for the pharmaceutical industry and the ethical questions about the use of animals for research (Van Norman, 2019).

*In silico* drug trials using human-based and biophysically-detailed models have proven to be a powerful technology for proarrhythmic risk predictions with high accuracy (Lancaster and Sobie, 2016; Passini et al., 2017; Li et al., 2019b; Passini et al., 2019; Llopis-Lorente et al., 2020). Their use has been promoted by regulators such as the United States Food and Drug Administration (FDA), that also launched the Comprehensive *in vitro* Proarrhythmia Assay (CiPA) initiative (Sager et al., 2014; Li et al., 2019a), and the European Medicines Agency (Musuamba et al., 2021), which established a task force on innovation for emerging therapies and technologies.

Integration of human-based *in silico* trials in drug safety assessment requires evaluating their consistency with experimental and clinical recordings used in safety pharmacology, such as Purkinje-based experiments. Therefore, the goal of this study is to quantify the electrophysiological effects of 14 reference drugs on a population of human-based *in silico* Purkinje models, to compare them with *in vitro* experiments in rabbit Purkinje preparations used preclinically in safety pharmacology, and to assess their accuracy for predictions of clinical proarrhythmic risk. The population of models approach (Britton et al., 2013; Muszkiewicz et al., 2015; Varshneya et al., 2021) scales the investigation from one single average model up to hundreds of models, to account for cell-to-cell electrophysiological variability and uncertainty. Therefore, with respect to the *in vitro* rabbit model, we hypothesized that human-based *in silico* drug trials will be overall consistent with experimental results but will improve predictions of drug-induced effects and clinical proarrhythmia risks, since they represent human pathophysiology and consider larger cellular variability in drug response.

## 2 Materials and methods

**FIGURE 1:**
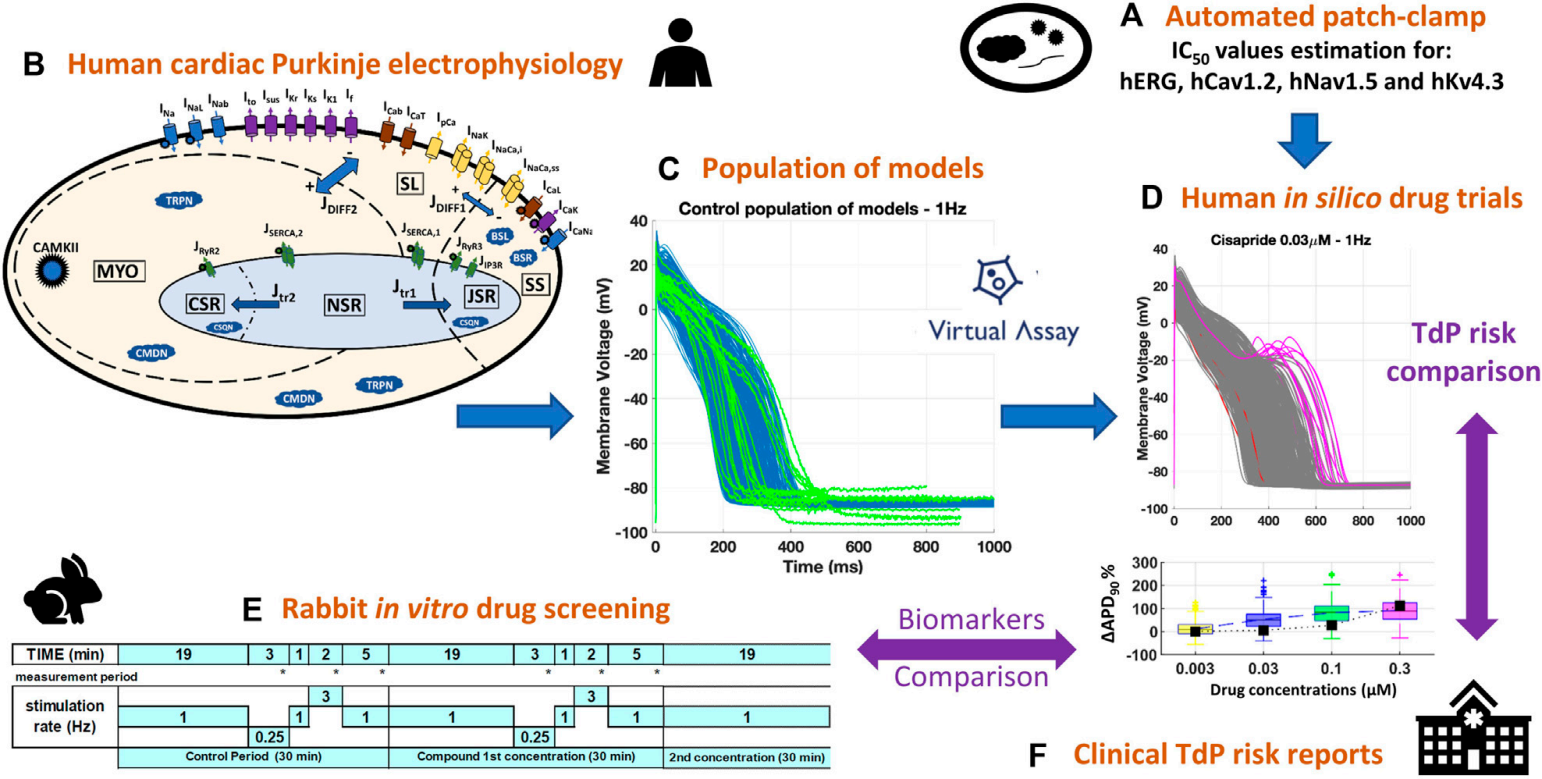
Combined experimental-computational pipeline used to perform this study. **(A)**: *In vitro* estimation of IC50s through automated patch clamp. **(B)**: Structure of the biophysically detailed computational model used to simulate human cardiac Purkinje electrophysiology (Trovato et al., 2020a). **(C)**: Experimentally-calibrated population of 530 models generated using the Virtual Assay software (Oxford University Innovation ^©^ 2018); blue traces: computational models; green traces: experimental AP recordings from human healthy cardiac Purkinje cell (Nagy et al., 2015). **(D)**: Representative example of human *in silico* drug trials on the population of models, including AP traces (with drug-induced repolarization abnormalities highlighted in pink) and biomarker boxplots. **(E)**: In silico results were compared against pre-existing *in vitro* recordings from rabbit Purkinje fibers, obtained with the protocol depicted for multiple frequencies and concentrations. **(F)**: Both *in vitro* and *in silico* results were compared against the clinical TdP risk from the CredibleMeds ^®^ repository (Woosley and Romer, 1999).

Figure 1 summarizes the methodology used in the present study, later described in more detail. Briefly, a diverse set of 14 reference compounds (Table 1) was investigated both *in vitro* and *in silico* to assess drug-induced changes in cardiac Purkinje electrophysiology and drug safety profiles. First, automated patch clamp was used to quantify the half-maximal inhibitory concentrations (IC50s) for four cardiac ion channels, for each compound (Figure 1A, Section 2.1.1). Then, simulations were conducted to investigate drug-induced electrophysiological changes in a population of 530 models based on a recent human cardiac Purkinje cell model (Figures 1B,C, Sections 2.2.1, 2.2.2). *In silico* predictions (Figure 1D, Section 2.2.3) were compared to preexisting voltage clamp recordings in rabbit Purkinje fibers (Figure 1E, Section 2.1.2) and clinical reports of Torsade de Pointes (TdP) arrhythmias (Figure 1F; Table 1). Different metrics (Section 2.3) were quantified from the *in vitro* and *in silico* assays to assess their consistency and ability to predict drug-induced AP changes, and clinical proarrhythmia risk as reported on the CredibleMeds^®^ repository (Woosley and Romer, 1999).

**TABLE 1:** List of reference compounds, IC50 values recorded for four ionic currents, tested concentrations in rabbit preparations, pacing frequencies, N^o^ of preparation used, and clinical proarrhythmic risk as reported by CredibleMeds ^®^ (Woosley and Romer, 1999).

Drug	IC50 (µM)				Tested concentration (µM) in rabbit preparations	Pacing frequencies (Hz)	N^o^ of rabbit preparations	TdP risk
	I_Na_	I_CaL_	I_to_	I_Kr_				
Astemizole	2.8	0.59	22	0.017	0.01, 0.1,1, 3	0.25, 1, 3	6	1
Bepridil	3.1	6	13	0.19	0.1, 0.3, 1, 3	0.2, 1, 3	6	1
Cisapride	—	33	—	0.015	0.003, 0.03, 0.01, 0.03	0.25, 1, 3	6	1
Clarithromycin	163^**^	103^**^	—	62.5^**^	1, 2.4, 10, 30	0.25, 1, 3	5	1
Diltiazem	15	0.76^*^	84	16.6	0.1, 1, 3, 10, 30	0.25, 1, 3	6	NC
Disopyramide	—	114	—	14.4*	0.3, 1, 3, 10, 30, 100	0.2, 1	5	1
Dofetilide	94	204	—	0.047	0.0003, 0.001, 0.003, 0.01	0.2, 1, 3	7	1
Nifedipine	23	0.051	31	92	0.03, 0.3, 1, 10	0.25, 1, 3	6	NC
Quinidine	35	2.9	15	1.26	0.1, 1, 3, 10	0.25, 1, 3	6	1
Ranolazine	101	156	—	24.5	0.3, 3, 10, 30	0.25, 1, 3	4	2
Risperidone	102	138	43	0.41	0.003, 0.03, 0.3	0.25, 1	6	2
Sotalol	—	—	—	86.4^**^	0.3, 1, 3, 10, 30	0.25, 1, 3	6	1
Terfenadine	3.3	2.2	68	0.17	0.03, 0.32, 1.44, 5.34	0.25, 1, 3	6	1
Verapamil	29	0.2*	58	0.6	0.1, 1, 3	0.25, 1, 3	6	NC

**IC50:** drug concentration leading to 50% of current inhibition. **I**_**Na**_: fast Na^+^ current; **I**_**CaL**_ **:** L-type Ca^2+^ current; **I**_**to**_: transient outward K^+^ current; **I**_**Kr**_: rapid delayed rectifier K^+^ current. **TdP risk**: Torsade the Points risk: 1) Known risk; 2) Conditional risk; NC) Not classified, i.e., evidence was not enough to add it to any risk category. Dashes indicate no effect or IC50 much higher than tested concentrations (corresponding to a negligible block of the current). *: from (Kramer et al., 2013); **: from (Crumb et al., 2016).

### 2.1 Experimental data

#### 2.1.1 *In vitro* ion channel data

The CiPA Ion Channel Work Group has investigated six cardiac ion channels of potential relevance for human QT effects. From those, we employed for this study four key human cardiac ion channels, including the minimum set of ion channels required for reliable *in silico* risk predictions (Zhou et al., 2020). This minimum set of ion channels includes the potassium channel hERG (modulating I_Kr_), the L-type calcium channel hCav1.2 (modulating the L-type Ca^2+^ current, I_CaL_), and the sodium channel hNav1.5 (responsible for the fast Na^+^ current, I_Na_). We also considered the potassium channel hKv4.3 (modulating the fast transient K^+^ current, I_to_) as IC_50_s were also available for this channel.

Experimental testing of ion channel inhibitions of hERG, hCav1.2, hNav1.5 and hKv4.3 was performed on engineered immortalized cell lines using patch clamp protocols at either room (21°C) or physiological (33°C) temperature. However, since ion channel inhibition data recorded at physiological temperature often show much greater inherent variability due to complexity of the protocol, only data generated at room temperature were considered throughout this study. Also, for the reference set of compounds, the room temperature protocol was found to better agree with published data (Crumb et al., 2016).

For *in vitro* hERG testing, hERG-CHO (Cytomyx CYL3038) were cultured in glutamax DMEM/F12 medium containing 10% fetal bovine serum and 1% G418 in a humidified, 5% CO2 atmosphere. Cells were grown at 37°C, then transferred to a 30°C incubator at least 24 h before the experiments to improve the hERG expression. HEK-293 cells were transfected stably with hERG cDNA and cultured in a 50:50 mix of Dulbecco’s Modified Eagle’s Medium and Ham’s Nutrient Mixture F-12 (DMEM/F-12) supplemented with 10% fetal bovine serum (FBS). A robotized planar patch clamp platform (HTX QPatch^®^ from Sophion, Denmark) was used, reproducing the experimental quality standards and analysis routines prevailing in manual patch clamp (giga-ohm seal, low access resistance, capacity compensation, run-down compensation as needed). The patch clamp protocol involved a −80 mV holding potential, then cells were depolarized to 20 mV for 5 s, followed by a 5 s deactivation pulse to −50 mV to reveal hERG tail current. This paradigm was delivered once every 15 s to monitor current amplitudes. Each concentration was tested in a standard protocol for at least 3 cells (*n* ≥ 3). Starting from −80 mV, effects of sample compounds on the onset and steady state inhibition of hERG current were followed in response to a repeated pulse pattern, damping from steady +20 mV to −80 mV. Obtained raw data was corrected for external effects at saturation concentration. Each compound was tested at least in triplicates at six appropriate concentrations in ascending order, and concentration limits were defined based on compound solubility, cytotoxicity, or compound physicochemical properties.

For the other ion channel targets (Cav1.2, Nav1.5 and Kv4.3) the screening was performed on the QPatch platform (Sophion, Ballerup, Denmark). HEK-293 and CHO (for Cav1.2) cell lines expressing exogenous human targets were cultured according to internal protocols, in DMEM/F12 media supplemented with 10% FBS. Standard 48 well plates were used in all experiments, and a standard voltage protocol mimicking elements of a ventricular action potential was applied at eight increasing concentrations to facilitate the determination IC50 values, with replicates. If no half-maximal inhibitory concentration was achieved in the specified concentration range, the result was interpreted as ‘no inhibition’. Missing or inconclusive data in our studies were complemented from literature (Kramer et al., 2013; Crumb et al., 2016; Passini et al., 2019). All IC50 values used to perform *in silico* trials are reported in Table 1.

#### 2.1.2 *In vitro* drug assay on rabbit purkinje fibers

The Purkinje fiber test assay is an advanced multicellular tissue model to determine the effects of a drug candidate on all ion channels that contribute to the formation of the cardiac action potential. The tissue offers good interpretability through its single cellular population expressing homogeneously all key ion channels. It is considered as a reliable surrogate model for QT prolongation effects and the *in vivo* ECG. The effects of the 14 reference compounds on cardiac Purkinje electrophysiology were previously evaluated through microelectrode recordings from isolated rabbit Purkinje fibers (male, New Zealand rabbits; 1.7–2.1 kg; 7–10 weeks of age). Details of the preparation and conduction of the studies have been previously described (Roche et al., 2010), though, in this study we consider new data, which were internally generated as part of the preclinical assessment of cardiac safety. Except for the studies of quinidine, risperidone and verapamil, no exclusion criteria were formulated in the study protocols. For those three compounds, exclusion criteria are identical to (Roche et al., 2010). No fiber was discarded based on the before-mentioned exclusion criteria.

In summary, test compounds were dissolved into dimethyl sulfoxide (DMSO) to obtain a stock solution. This solution was further diluted into 100% DMSO to obtain solutions at different concentrations (as listed in Table 1) and added into the physiological solution. The Purkinje fibers (5-7 per compound, as detailed in Table 1) were first superfused with an oxygenated physiological solution containing (in mmol/L): NaCl 120; KCl 4; MgCl2 1; NaH2PO4 1.8; NaHCO3 25; glucose 11; CaCl2 1.8; pH = 7.4, at 36 ± 1°C. After a 30-min control period, the test compound was evaluated at increasing concentrations that were sequentially applied, every 30 min, on a specific subset of preparations as reported in Table 1. For the control period and each tested concentration, the fibers were stimulated at the basal rate of 1 pulse per second (1 Hz, normal pacing rate). In addition, the stimulation rate was decreased from 1 pulse per second (1 Hz) to 1 pulse every 4 or 5 s (0.25 or 0.2 Hz, as specified in Table 1, low pacing rate) for 3 min, returned to 1 pulse per second for 1 min and then increased to 3 pulses per second (3 Hz, high pacing rate) for 2 additional minutes (between the 19th and the 25th minute) and finally decreased to 1 pulse per second (1 Hz) from the 25th to 30th minute as illustrated in Figure 1E.

The following biomarkers were quantified: take-off potential (TOP, in mV), i.e., the membrane voltage preceding phase 0, AP amplitude (APA, in mV), maximal upstroke velocity (dV/dt_MAX_, in V/s), AP duration at 50% and 90% of repolarization (APD_50_ and APD_90_ in ms).

The low stimulation rate favors the occurrence of abnormal electrical events during the repolarization phase of the action potential, such as early after depolarizations (EADs). After testing the highest concentration, the physiological solution was superfused again to evaluate the reversibility of the drug effect.

### 2.2 Human *in silico* drug trials

#### 2.2.1 *In silico* human cardiac purkinje model

Human cardiac Purkinje electrophysiology was simulated using the Trovato2020 *in silico* model (Trovato et al., 2020a), publicly available on the model repository of the Computational Cardiovascular Science Team (www.cs.ox.ac.uk/insilicocardiotox/model-repository). As shown in Figure 1B, the main ionic currents of this model are fast and late Na^+^ currents (I_Na_ and I_NaL_, respectively), I_CaL_, T-type Ca^2+^ current (I_CaT_), I_to_, sustained outward K^+^ current (I_sus_), rapid and slow delayed K^+^ rectifiers (I_Kr_ and I_Ks_, respectively), inward K^+^ rectifiers (I_K1_), funny current (I_f_), Na^+^-Ca^2+^ exchanger (I_NCX_) and Na^+^-K^+^ pump (I_NaK_). The model was calibrated and evaluated against a wide set of AP recordings from human healthy Purkinje cells in control conditions and after exposure to dofetilide, and it is also able to reproduce the most common arrhythmia precursors at the cellular level, i.e., early and delayed afterdepolarization (EADs and DADs, respectively).

#### 2.2.2 Population of human cardiac purkinje models

Starting from the Trovato2020 model, we developed a virtual population of human cardiac Purkinje cells, to incorporate biological variability. The population was designed similarly to previous work in (Trovato et al., 2020b), and using the well-established population of models methodology (Britton et al., 2013; Muszkiewicz et al., 2015). All simulations were performed using the Virtual Assay software (v.3.2 ^©^ 2018 Oxford University Innovation Ltd. Oxford, UK), a user-friendly software to perform *in silico* drug trials in population of models (Passini et al., 2021). An initial population of 1,000 models was constructed by sampling the 12 main ionic current conductances mentioned above (I_Na_, I_NaL_, I_CaL_, I_CaT_, I_to_, I_sus_, I_Kr_, I_Ks_, I_f_, I_K1_, I_NCX_, I_NaK_) in the range [50–200]% of their baseline values, using Latin hypercube sampling (McKay et al., 1979). First, models were paced individually for 1,000 beats to allow relaxation from the initial conditions and to reach the steady state at normal pacing (1 Hz). For each model, nine AP biomarkers were computed on the last simulated beat, including the experimental biomarkers described above (APD_90_, APD_50_, dV/dt_Max_, TOP and APA), and additionally the AP duration at 10%, 25%, and 75% of repolarization (APD_10_, APD_25_, APD_75_) and the “end of potential” voltage (EOP). Only models exhibiting all AP biomarkers within the experimental ranges measured in healthy human Purkinje cells (Nagy et al., 2015; Trovato et al., 2020a) and no repolarization abnormalities (i.e., EADs or DADs) were retained in the calibrated population. All models in the calibrated population were also paced for further 150 beats at slow pacing (0.2 or 0.25 Hz) and fast pacing (3 Hz). This was done to exclude models showing repolarization abnormalities also at these pacing frequencies in control and to obtain control AP biomarkers for all the frequencies used in the *in vitro* experiments. The final calibrated population consists of 530 models and all the drug trials were performed exactly on the same population with no further modifications.

### 2.3 Human *in silico* drug trials

**FIGURE 2:**
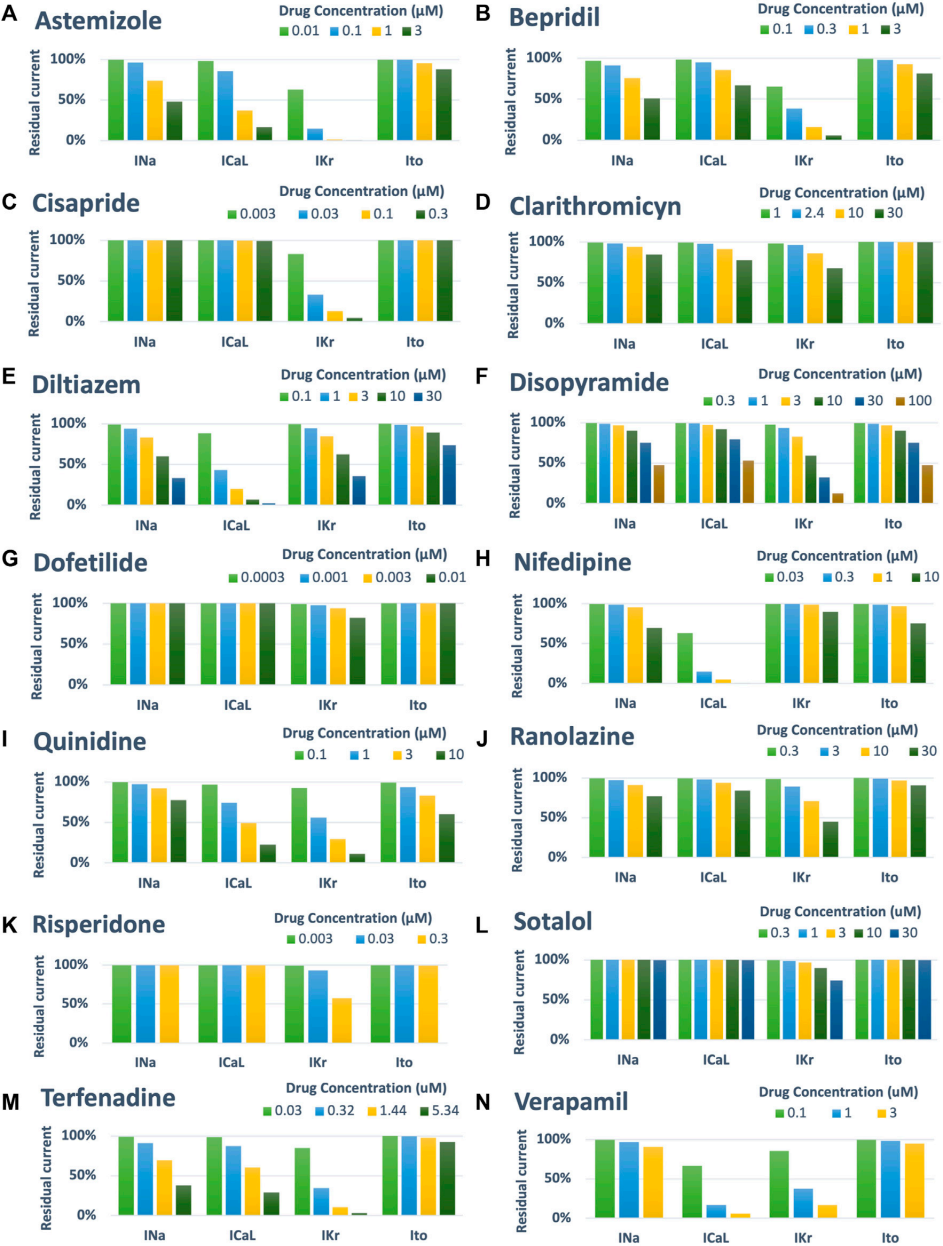
Summary of drug-induced effects on the cardiac ion channels, computed with a simple pore-block model. Each panel shows one of the fourteen reference compounds, with the different bars representing the residual current after drug-application (in percentage), for each ion channel and drug concentration.

Drug-induced inhibition of the different ion channels was simulated using a simple pore-block model (Brennan et al., 2009), with the experimental IC50 and drug concentrations reported in Table 1 for I_Na_, I_CaL_, I_Kr_ and I_to_, and Hill coefficients equal to 1. Figure 2 shows a visual representation of the residual currents following drug administration, for each compound and each concentration.

Starting from the steady state described above, the models were paced for further 150 beats at each frequency including the drug effects. Extracellular concentrations were set as in the *in vitro* rabbit experiments, and the same AP biomarkers were computed on the last simulated beat. Repolarization abnormalities were detected as positive derivatives of the membrane voltage over time, occurring after 150 s, as in (Trovato et al., 2020b). AP biomarkers were not computed for models showing abnormalities.

All simulations were performed on a regular Desktop Computer (Intel (R) Core (TM) i5-4670S CPU @ 3.10 GHz RAM: 8 GB, 64-bit Windows 10). The time required to simulate one drug at one concentration (150 beats) in a population of 530 models was 12, 17, and 35 min, for simulations at 3, 1, and 0.2 Hz, respectively.

### 2.4 Metrics for comparison of experiments, simulations, and clinical evidence

We first compared the mean of simulated and experimental AP biomarkers in control conditions (no drug) for all pacing frequencies, to assess the consistency between the *in silico* and *in vitro* models. Then, we benchmarked the results for each compound at each concentration and pacing frequency against control. Results are shown as percent variations of the means, for both experiments and simulations.

For proarrhythmia risk assessment, we considered two different metrics. The first is the occurrences of drug-induced abnormalities, as previously used by (Passini et al., 2017). In summary, compounds inducing repolarization abnormalities in at least one model of the *in silico* population or 1 cell of the *in vitro* rabbit assay, at any of the tested concentrations, were classified as risky, whereas they were classified as safe if all models/cells fully repolarized at slow pacing. However, differently from (Passini et al., 2017) we used a slower pacing frequencies (0.2 or 0.25 Hz, depending on the compound as reported in Table 1) to mimic the same experimental conditions as for the *in vitro* rabbit assay.

The second metric is based on APD_90_ prolongation, since it is one of the most common biomarkers to discriminate between safe and proarrhythmic compounds *in vitro*, though some limitations have been observed (Redfern et al., 2003; Champeroux et al., 2005). In accordance with experimental thresholds applied in preclinical tests, we considered a mean APD_90_ prolongation higher than 10% as a warning for possible drug-induced proarrhythmic effects. Classification results based on these metrics for *in vitro* rabbit and *in silico* human trials were then compared against the clinical risk as reported by CredibleMeds ^®^ (Woosley and Romer, 1999), which divides drugs in multiple categories, based on TdP risk. As shown in Table 1, the 14 reference compounds match either category 1 (high risk: the drug prolongs the QT interval and is clearly associated with a known proarrhythmia risk, even when taken as recommended), 2 (conditional risk: the drug is associated with TdP but only under certain circumstances, e.g., overdose or interaction with other drugs), or NC (not classified - the drug was reviewed by CredibleMeds^®^ but the evidence available was not enough to assign it to any of the previous categories. Here, drugs in categories 1 and 2 were considered risky, while drugs in category NC were considered safe.

Finally, to evaluate the consistency between experiments and simulations, we applied a third metric, based on the mean of drug-induced percent variations in AP biomarkers for each tested drug and concentration: 1) strong agreement, if the trend (increase/decrease) was the same, and the difference between the *in vitro* and *in silico* means was equal or less than 15%; 2) qualitative agreement, if the trend (increase/decrease) was the same, but the difference between the *in vitro* and *in silico* means higher than 15%; 3) disagreement, if the trend was different, regardless of the magnitude of the mean difference.

## 3 Results

### 3.1 Simulated and experimental AP biomarkers in control

Table 2 reports mean and standard deviation of each AP biomarker in control conditions for the *in vitro* rabbit experiments (*n* = 74) and the population of human *in silico* models (*n* = 530) at slow, normal, and fast pacing. APD_90_ (and to a lesser extent APD_50_) is larger in rabbit experiments compared to human simulations at all pacing frequencies and, to a greater extent, at slow pacing. Decreasing the pacing frequency results in larger APD prolongation in rabbit than human Purkinje cells, in agreement with what has been previously measured in rabbit (Li et al., 2016) and human (Nagy et al., 2015) Purkinje cells. Both dV/dt_MAX_ and APA were also larger in rabbit experiments than human simulations: again, this is in agreement with known inter-species differences (Cohen et al., 1984; Roche et al., 2010; Nagy et al., 2015). No major differences were observed for TOP.

**TABLE 2:** Experimental and simulated AP biomarkers in control conditions (no drug) at slow pacing (0.25), normal pacing (1 Hz) and fast pacing (3 Hz).

Control						
	Slow pacing		Normal pacing		Fast pacing	
	Rabbit *in vitro*	Human *in silico*	Rabbit *in vitro*	Human *in silico*	Rabbit *in vitro*	Human *in silico*
APD_90_	432 ± 119	292 ± 65	310 ± 52	281 ± 55	216 ± 24	212 ± 27
APD_50_	311 ± 107	215 ± 59	239 ± 53	210 ± 50	162 ± 24	149 ± 22
dV/dt_Max_	627 ± 80	363 ± 73	616 ± 72	419 ± 79	595 ± 62	391 ± 79
APA	128 ± 3	107 ± 4	129 ± 3	112 ± 4	129 ± 3	113 ± 4
TOP	-89 ± 1	-84 ± 1	-91 ± 1	-87 ± 1	-92 ± 1	-88 ± 1

Data shown as mean ± standard deviation. Rabbit *in vitro*: experiments in *in vitro* rabbit cardiac Purkinje fibers (*n* = 74); Human *in silico*: simulations in human cardiac Purkinje AP, models (*n* = 530). APD_X_: AP, duration at X% of repolarization; dV/dt_MAX_: maximal upstroke velocity; APA: AP, amplitude; TOP: take-off potential.

### 3.2 Proarrhythmic risk assessment based on drug-induced repolarization abnormalities

**FIGURE 3:**
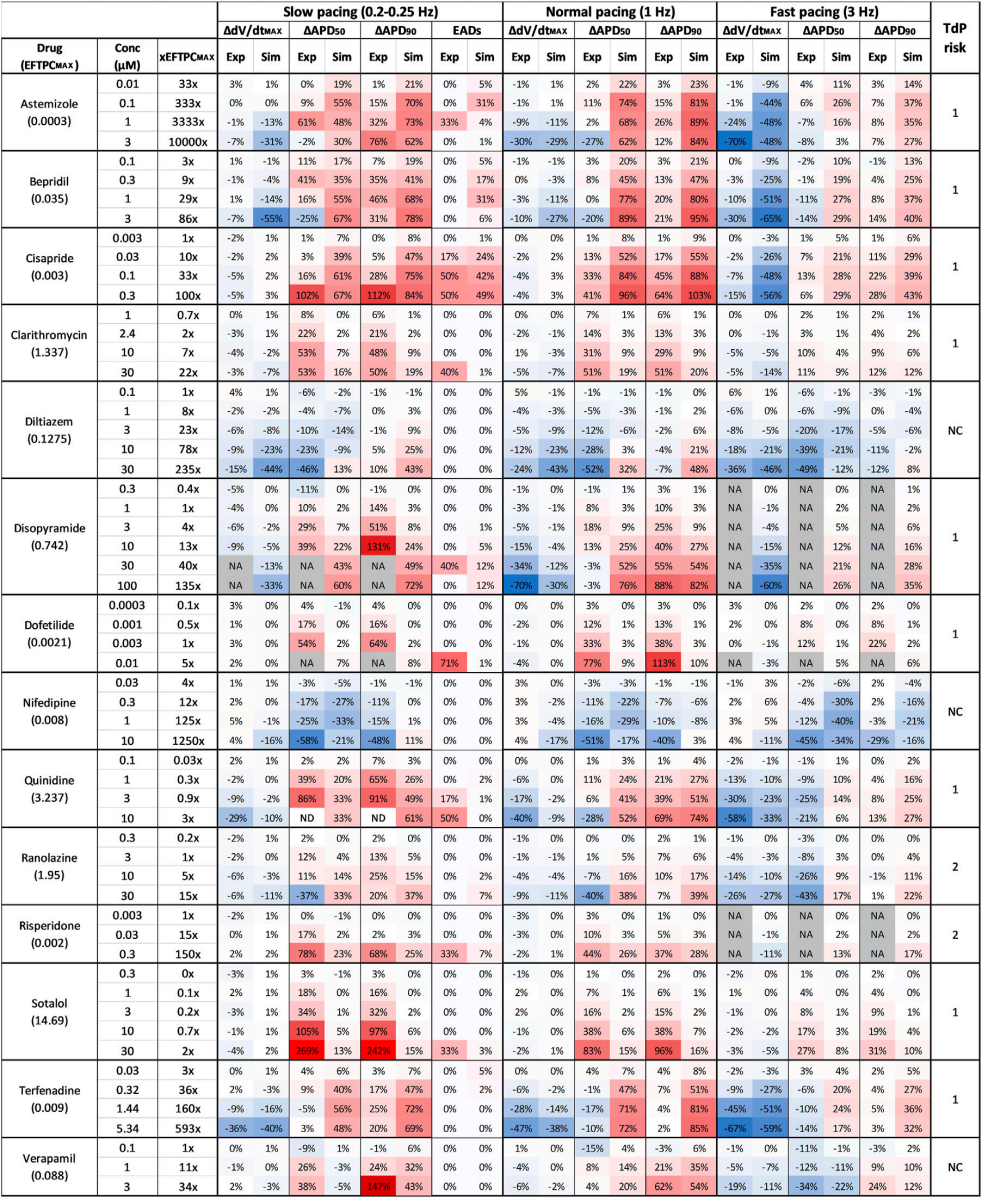
Comparison between clinical TdP risk and percent drug-induced AP changes in human simulations and rabbit experiments for each drug, at different concentrations and pacing frequencies. Columns description from left to right: first: drug name and maximum effective free plasma concentrations (EFTPC_MAX_, in µM); second: tested drug concentrations; third: ratio between tested concentrations and EFTPC_MAX_; from fourth to ninth: experimental and simulated AP biomarkers changes at slow pacing; 10th to 11th: percentage of EADs occurrences in simulations and experiments at slow pacing; from 12th to 23rd: experimental and simulated AP biomarkers changes at normal and fast pacing; 24th: drug risk category based on CredibleMeds ^®^ (Woosley and Romer, 1999): high risk (1), conditional risk (2), NC (non-classified in any risk category). NA: data not available. Changes are shown as percentage with respect to control conditions. Colors indicates biomarker increase (red) or decrease (blue) and are scaled from the minimum to the maximum percent variations for each biomarker at each pacing frequencies.

Figure 3 summarizes drug-induced electrophysiological percent changes for both rabbit *in vitro* recordings and human *in silico* simulations, at slow, normal, and fast pacing. Changes are reported for the most informative AP biomarkers, namely dV/dt_Max_, APD_50_ and APD_90_, as negligible changes were observed for TOP in both rabbit recordings and human simulations, and changes in APA showed the same trend as for dV/dt_Max_. Also, EADs occurrences are reported for both experiments and simulations only at slow pacing, since no rabbit *in vitro* EADs screening was performed at normal and fast pacing. Results are shown for each compound at each concentrations reported in Table 2 and expressed also as a multiple of the maximum effective free therapeutic plasma concentrations (EFTPCmax) reported in the literature (Kramer et al., 2013; Crumb et al., 2016; Passini et al., 2019).

**FIGURE 4:**
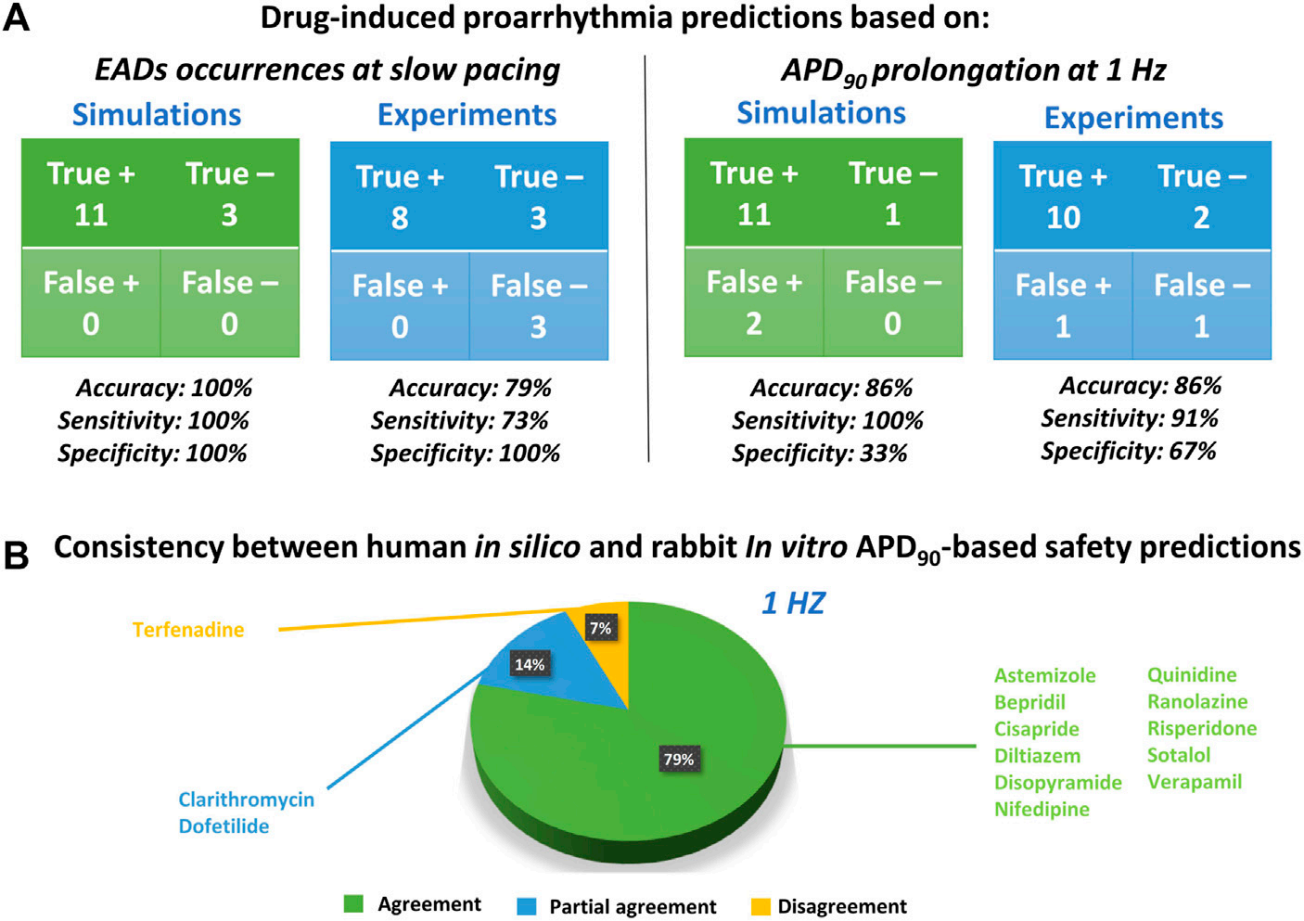
Proarrhythmic risk predictions using *in silico* human or *in vitro* rabbit models. **(A)** Confusion matrix for *in silico* (green) and *in vitro* (blue) predictions, compared to clinical report of proarrhythmia, based on EADs occurrences (left panel) or APD_90_ (right panel); +: Risky drug; -: Safe drug. **(B)** Consistency between *in silico* and *in vitro* drug safety predictions based on APD_90_ prolongation at 1 Hz. The pie chart represents the percentage of compounds showing agreement for most of the concentration tested (green), partial agreement for at least half of the tested concentrations (blue), or disagreement (yellow).

The occurrence of drug-induced abnormalities in repolarization was quantified in both experiments and simulations as reported in Figure 3 (11th and 12th columns, respectively). These data were used to classify drugs as safe or risky, as previously described (Section 2.3), and the results are shown in Figure 4A (left panel). Human *in silico* drug trials correctly classified all drugs (accuracy = 100%), while the *in vitro* rabbit models achieved an accuracy of 79%. Indeed, in rabbit preparations, EADs were observed for only 8 out of 11 risky compounds, while no EADs were observed for bepridil, ranolazine, and terfenadine, despite the wide range of concentrations explored. Diltiazem, nifedipine and verapamil did not induce any EADs *in silico* nor *in vitro* and were correctly classified as safe.

**FIGURE 5:**
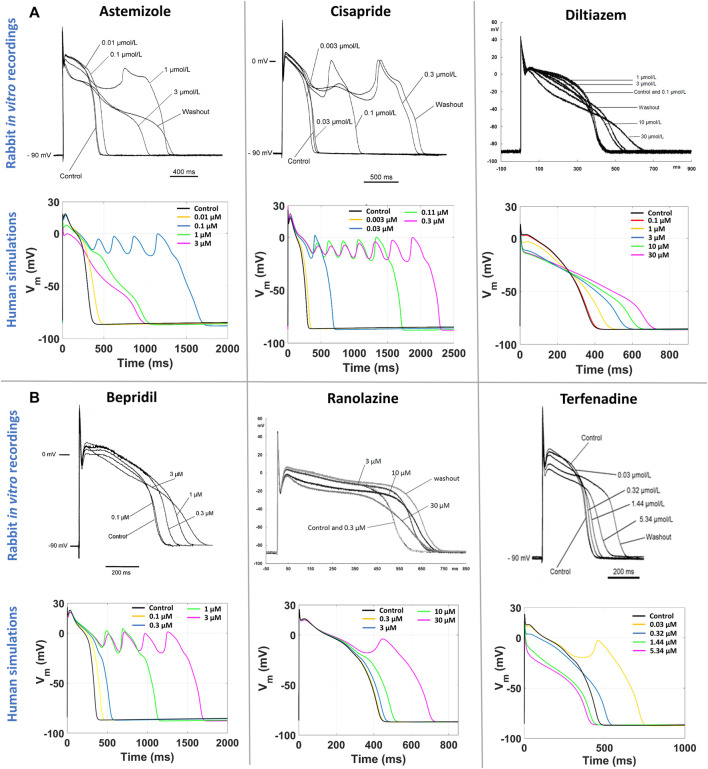
Comparison between human *in silico* and *in vitro* rabbit AP traces at slow pacing, for six illustrative compounds. In each section, experimental recordings are at the top, and simulated traces at the bottom. One representative cell/model is shown for each drug. **(A)** Three explicative compounds showing EADs in both simulations and experiments: astemizole (left), cisapride (center), diltiazem (right). **(B)** The three compounds showing disagreement in EADs occurrence between *in silico* and *in vitro* results, i.e., EADs were observed only in simulations and not in experiments: bepridil (left), ranolazine (center), terfenadine (right).

Figure 5A reports a comparison between experimental and simulated AP traces for three illustrative compounds. Astemizole and cisapride (Figure 5A, left and central panels respectively) induced EADs in both simulations and experiments. For astemizole, EADs occurred at lower concentrations and largely disappeared at higher concentrations, due to the concurrent strong (>50%) inhibition of I_CaL_ (Figure 2, panel A), whereas for cisapride EADs were observed up to the highest tested concentration, with increasing duration. Diltiazem (Figure 5A, right panel) lowered the AP plateau and increased the AP duration in both experiments and simulations, but it did not induce any repolarization abnormalities, in line with its safe profile.

Figure 5B shows experimental and simulated AP traces for the three risky compounds that were correctly identified by the human simulations based on EADs occurrence (bottom panels), but misclassified by the rabbit experiments, i.e., bepridil, ranolazine and terfenadine. Simulations with bepridil showed EADs duration increasing with drug concentrations, similarly to cisapride, despite a mild (<50%) inhibition of I_CaL_ (Figure 2, panel B). Simulations with ranolazine showed EADs only at the highest concentration assessed (Figure 5B, central panel), in line with its conditional proarrhythmic profile, i.e., risk of inducing TdP only under certain circumstances (e.g., overdose, in patients with concurrent conditions such as hypokalemia) or by creating conditions that facilitate TdP (Woosley and Romer, 1999). Finally, simulations of terfenadine (Figure 5B, right panel) displayed EADs only at lower concentrations, similarly to astemizole. In this experiment, compound stickiness may have led to tubing absorption for this compound, leading to lower effective concentration at the higher dosing concentrations, though, no documented evidence is available.

### 3.3 Proarrhythmia risk assessment through AP prolongation: Consistency between experiments, simulations, and clinical reports

Since APD_90_ is widely used in preclinical safety studies, we also evaluated drug-induced changes in APD_90_ in rabbit experiments and human simulations. Figure 4A (right panel) shows the confusion matrices obtained by considering as risky drugs showing at 1 Hz average APD_90_ prolongation greater than 10%, as described in Section 2.3. Based on this metric, human *in silico* drug trials correctly classified all risky drugs, even though two safe drugs resulted in false positives, yielding a total accuracy of 86%. This can be explained by the fact that diltiazem and verapamil showed significant AP prolongation at high concentrations (78x and 11x, respectively) and were therefore classified as risky. This AP prolongation is due to the large (>50%) hERG blockade at concentrations far from the EFTPCmax. *In vitro* rabbit assays produced the same overall accuracy, even though it was achieved with one false positive (verapamil) and one false negative (terfenadine), the latter showing little AP prolongation at all tested concentrations tested (up to 593x EFTPC_MAX_), despite its high TdP risk.

The pie chart in Figure 4B summarizes the consistency between *in silico* and *in vitro* predictions based on APD_90_ prolongation at 1 Hz: out of 14 reference compounds, 11 were in agreement, 2 were in partial agreement, and only 1 was in disagreement (categories defined as described in Section 2.3). Disagreement was observed for terfenadine, which induced up to 85% AP prolongation in simulations, and only up to 7% in *vitro* rabbit experiments.

Partial agreement was observed for clarithromycin and dofetilide. As shown in Figure 3, both *in silico* and *in vitro* results show dose-dependent AP prolongation for both drugs. However, the changes observed in rabbit experiments were larger than those observed in simulations (at the maximum tested concentration: 113% vs. 10% for dofetilide in rabbit experiments and human simulations, respectively; 51% vs. 20% for clarithromycin). A similar behavior was also observed for sotalol (Figure 3), and it can be related to known species differences between humans and rabbit in the response to hERG blockers (Lu et al., 2001; Lu et al., 2002), i.e. larger drug-induced AP prolongation in rabbit as detailed below (Section 4). It is also worth to notice that *in vitro* experiments for dofetilide and sotalol were performed in a narrow range of concentrations (up to 5x and 2x the EFTPCmax, respectively), limited by the solubility of the drugs. This limitation can be easily overcome with *in silico* trials, and–when simulating higher concentrations–we observed larger AP prolongations in the population of models: +57% for dofetilide 0.1 µM and +39% for sotalol at 100 µM.

### 3.4 Detailed comparison of human *in silico* and rabbit *in vitro* drug trials

**FIGURE 6:**
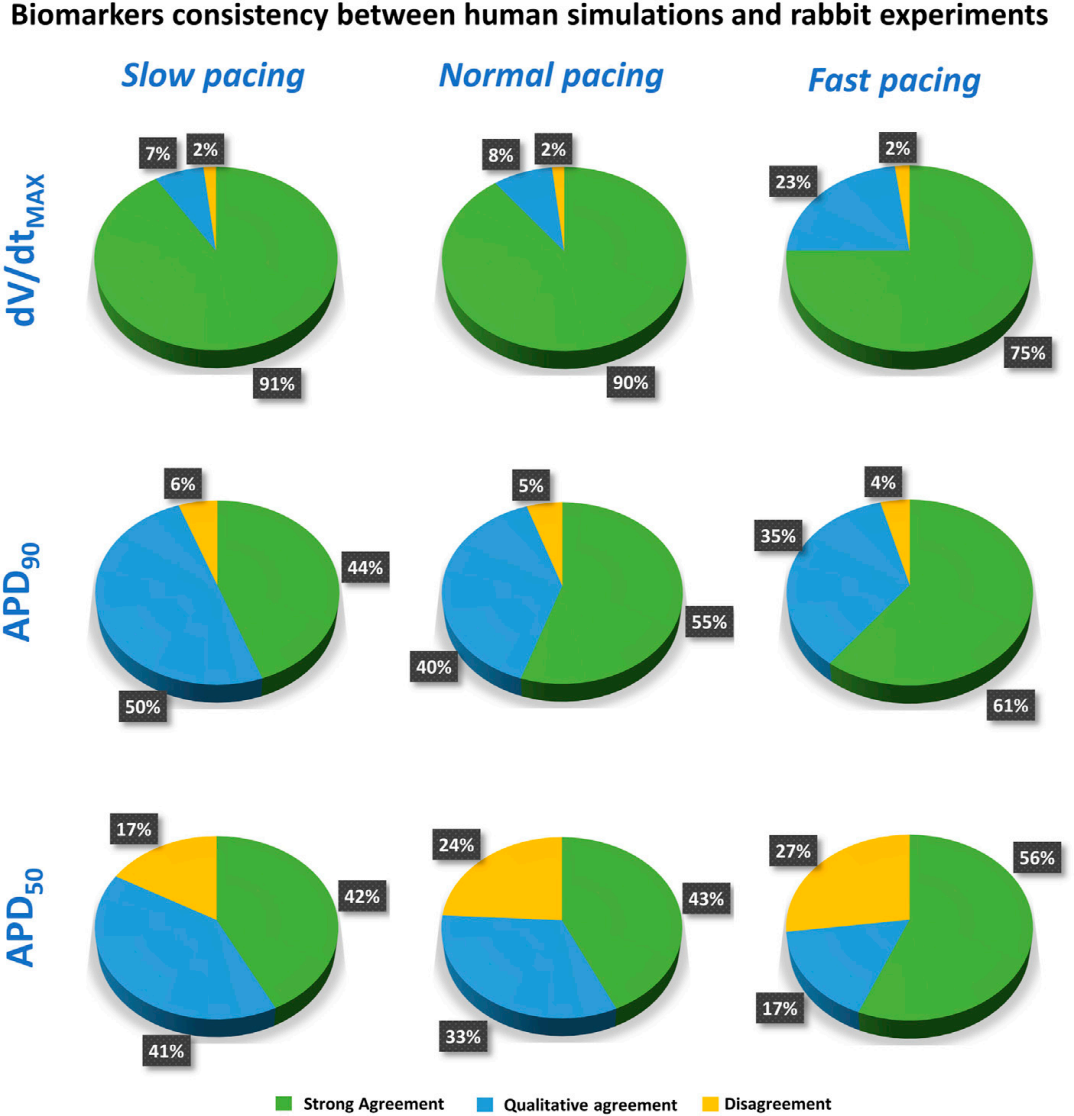
Summary of the comparison between experiments and simulations at different pacing frequencies, for dV/dt_MAX_ (top), APD_90_ (middle), and APD_50_ (bottom). Pie charts show the percentage of tested compounds at different concentrations in strong agreement (green), qualitative agreement (blue), or disagreement (yellow). Categories defined as in Section 2.3).

Figure 6 presents the comparison between experimental and simulation results, based on the results from Figure 3 and using the AP biomarkers: each pie chart includes (for each biomarker, at each pacing frequency) the percentage of drug concentrations in either strong agreement (green), qualitative agreement (blue), or disagreement (yellow), across all drug trials. These results clearly demonstrate a high degree of consistency between experiments and simulations, across all drugs, concentrations, pacing frequencies, and AP biomarkers.

Results for dV/dt_MAX_ show an almost total agreement (98% for all pacing frequencies): the only drug showing disagreement was nifedipine at 10 μM, which slightly increased dV/dt_MAX_ (4%) in rabbit and reduced it in humans (−16%, −17% and −11% at slow, normal, and fast pacing, respectively). Drug-induced APD_90_ changes also showed a high degree of consistency (94–96%) between experiments and simulations, even though - compared to dV/dt_MAX_ - a larger portion of drugs showed qualitative rather than strong agreement. Disagreement was only found in 6%, 5%, and 4% of drug concentrations, for slow, normal, and fast pacing, respectively. Results for APD_50_ also showed good agreement (73–83%), despite the percentage of disagreement being higher compared to dV/dt_MAX_ and APD_90_. This difference could be explained by the differences in AP morphology between humans and rabbit: rabbit APs have a more pronounced spike compared to human *in silico* models and this affected the voltage threshold to compute the APD_50_ (Figure 5).

**FIGURE 7:**
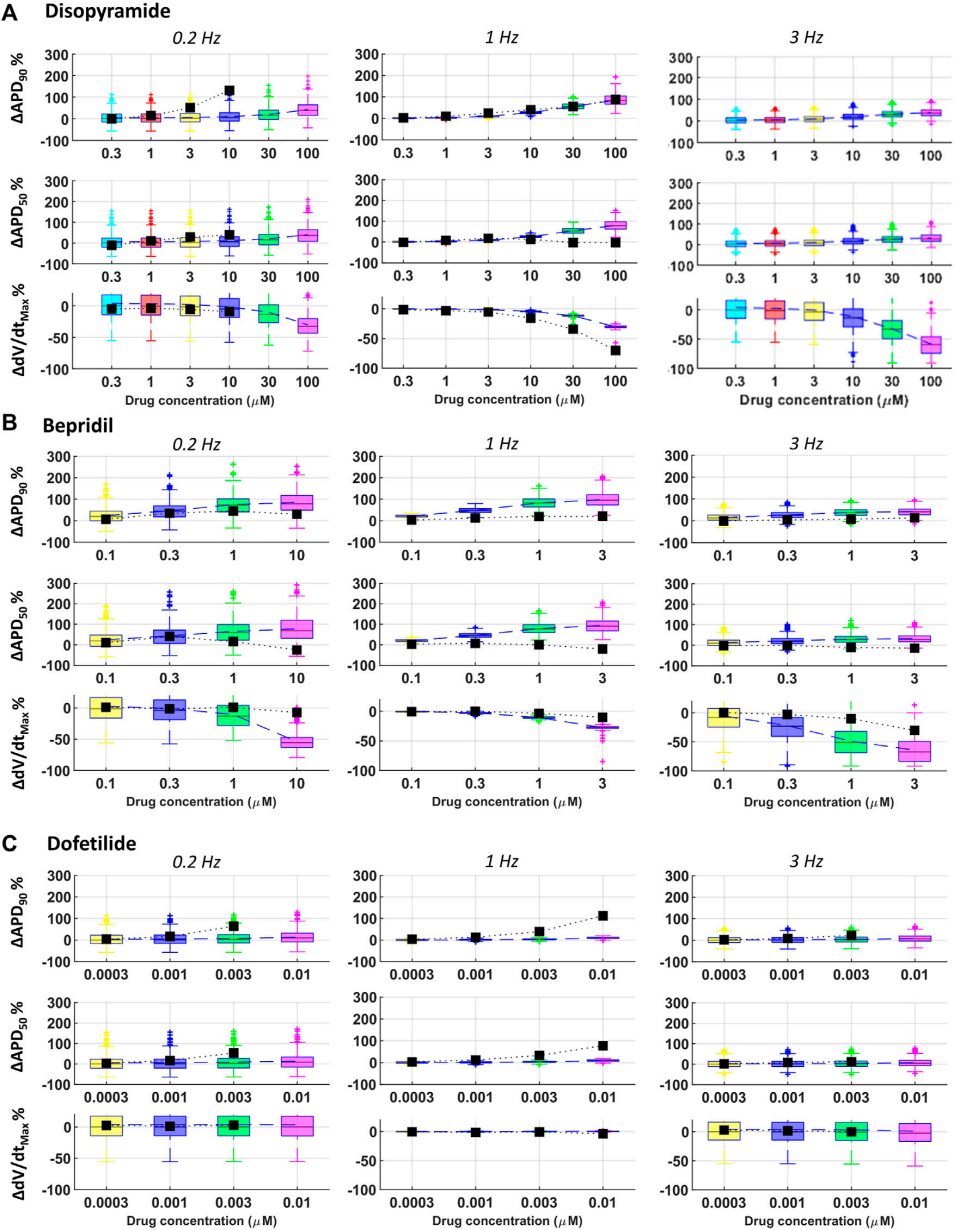
Comparison between simulated and experimental dose-response curves for APD_90_, APD_50_ and dV/dt_MAX_, for three representative compounds, at all pacing frequencies: **(A)** disopyramide; **(B)** bepridil; **(C)** dofetilide. Boxplots: human simulations at different concentrations (one color per concentration): on each box, the central mark is the median of the population, box limits are the 25 and 75th percentiles, and whiskers extend to the most extreme data points not considered outliers, plotted individually as separate crosses. Black squares: mean values of the *in vitro* rabbit data.

Disopyramide showed larger APD_90_ at slow pacing in rabbit experiments compared to simulations, and smaller APD_50_ at 1 Hz and high concentration (Figure 7A).

Results for quinidine and risperidone were strongly consistent at 1 Hz, whereas - at slow pacing - a larger AP prolongation was observed in experiments compared to simulations (Figure 3). This could be due to differences in rate-dependent drug-induced effects on I_CaL_ and I_Kr_ between human and rabbit as reported in (Nagy et al., 2015; Li et al., 2016).

For all tested concentrations, astemizole, bepridil (Figure 7B) and cisapride induced a larger increase in APD_90_ in simulations compared to rabbit preparations, especially at 1 Hz and remarkably high concentrations. For these three compounds, dV/dt_MAX_ predictions were strongly in agreement with the experiments at 1 Hz, whereas, at both slow and fast pacing, they led to a larger reduction in dV/dt_MAX_ in simulations than experiments. For astemizole and bepridil, APD_50_ was the less consistent biomarker, especially at high frequency, since simulations showed marked APD_50_ increased which was not observed in experiments. Simulations and experiments for clarithromycin showed agreement at fast pacing, whereas at slow pacing, simulations were just qualitatively in agreement, showing smaller AP prolongation compared to experiments. Simulations and experiments for ranolazine and terfenadine agreed for dV/dt_MAX_ at every pacing frequency and qualitatively for APD_90_ at slow pacing. At higher frequencies human simulations showed AP prolongation larger than in rabbit experiments. Simulated drug induced APD_90_ and APD_50_ prolongations for dofetilide (Figure 7C) and sotalol were significantly smaller than those observed experimentally in rabbit, at all tested concentrations and pacing frequencies, due to well-known species differences in hERG block sensitivity as discussed in Section 3.3.

## 4 Discussion and conclusion

In this study, we showcase the large impact that human *in silico* drug trials can have in the context of predictions of drug-induced proarrhythmic risk based on ion channel information, by providing new evidence obtained through simulations in human cardiac Purkinje cells. We present results for a selection of 14 reference compounds (at multiple concentrations and pacing rates), using *in silico* human Purkinje models with a variety of ionic profiles (*n* = 530), their comparison to preexisting *in vitro* rabbit Purkinje fibers experiments (4 ≤ *n* ≤ 6 for test compound), and their ability to predict clinical risk, based on various biomarkers, including EADs occurrence and APD_90_.

The main findings of this study are:

1 *In silico* predictions using human Purkinje models based on EAD occurrence at slow pacing showed 100% accuracy in classifying risky from safe drugs, while *in vitro* rabbit experiments yielded 79% accuracy. This was also superior to predictions based on AP prolongation, which yielded accuracy of 86% for both *in silico* and *in vitro.*
2 *In silico* drug trials using human cardiac Purkinje electrophysiology models and *in vitro* rabbit Purkinje recordings showed a high degree of consistency for all tested compounds, across biomarkers, concentrations and pacing frequencies. This supports the credibility of human-based *in silico* modelling and simulations for the replacement of animal experiments in this context of use.
3 Three compounds, i.e., clarithromycin, dofetilide, sotalol, displayed a larger AP prolongation *in vitro* rabbit compared to *in silico* human recordings. This agrees with well-known differences between rabbits and humans in the response to hERG block.

The high translatability of human-based *in silico* drug trials to clinical outcome - as demonstrated here for electrophysiology–highlights their potential for high regulatory impact in drug discovery (Musuamba et al., 2021). Human-based computational simulations can accurately predict clinical drug-induced arrhythmia (Lancaster and Sobie 2016; Passini et al., 2017; Li et al., 2019b; Passini et al., 2019; Llopis-Lorente et al., 2020). This is particularly relevant for compounds with positive hERG assays that may not induce arrhythmia due to their concomitant effect on I_Na_ and I_CaL_. Several studies have focused only on hERG signals for TdP risk predictions (Leishman et al., 2020; Ridder et al., 2020), whereas here we used IC50s for four ion channels, and mechanistic modelling and simulation of cardiac cellular electrophysiology. Therefore, drugs such as verapamil, that have a strong hERG block but low risk of TdP due to the concomitant Cav1.2 inhibition, are correctly identified as safe through the human-based modelling and simulation framework presented in this study. We previously demonstrated how human *in silico* trials using ventricular cardiomyocytes reach higher accuracy than animal models for proarrhythmia risk prediction (Passini et al., 2017), and also their consistency with recordings from isolated rabbit wedge and calcium transients from human induced pluripotent stem cell-derived cardiomyocytes (hiPSC-CMs). Here we compare proarrhythmic risk predictions, drug-induced electrophysiological changes in human *in silico* cardiac Purkinje cells and preexisting *in vitro* AP recordings from rabbit Purkinje fibers, which were previously produced for preclinical safety pharmacology assessments. Therefore, no further animal experiments were performed to realize this study as we used as much existing data as possible, with obvious benefits in terms of the 3Rs (replace, refinement, reduction) for animal research (Burden et al., 2015).

Also, in this study we decided to compare human *in silico* predictions with rabbit *in vitro* observations, i.e., heterogenous data as we are comparing two different methodologies and two different species. Two reasons underly this choice: 1) rabbit Purkinje fibers are commonly used as an *in vitro* model to predict proarrhythmic risk in human. Therefore, here we evaluate how the human-based *in silico* methodology performs compared to commonly used experimental models. This is particularly relevant for the 3Rs, as it allows reusing data. 2) the ultimate goal of preclinical studies is to predict proarrhythmic risk in humans, and human-based models that capture human patho-physiology are crucial to overcome some of the limitations of animal models. Using combinations of experimental and computational methods effectively require the evaluation of their consistency as performed here.

For preclinical risk assessment, we first considered a metric based on the occurrences of drug-induced repolarization abnormalities at slow pacing, similar to (Sager et al., 2014; Passini et al., 2017; Varshneya et al., 2021). We reached an accuracy of 100% using human *in silico* drug trials, compared to only 86% using *in vitro* recordings, which failed to identify two compounds with known TdP risk (bepridil and terfenadine) and one with conditional TdP risk (ranolazine). Our findings agree with previous experimental studies, showing that these three compounds often did not show EADs when tested in rabbit preparations. Bepridil up to 10 µM did not induce EADs in rabbit hearts (Anno et al., 1984; Hondeghem et al., 2003), while it did on hiPSC-CMs at the same concentration (Yu et al., 2019). Similarly, terfenadine did not induce EADs in rabbit wedge preparations (Liu et al., 2006; Vos, 2008), but it did in hiPSC-CMs (Nozaki et al., 2014). Ranolazine, which is associated with TdP only under certain conditions, e.g. hypokalemia, bradycardia, etc. (Woosley and Romer, 1999), has shown anti-torsadogenic effects in rabbit hearts at 10 µM (Frommeyer et al., 2012; Sossalla et al., 2014), but at 100 µM induced EADs on hiPSC-CMs (Blinova et al., 2018; Yu et al., 2019).

From a mechanistic point of view, models developing EADs were characterized by high conductances for depolarizing currents as I_NaL_ and I_CaL_ and low conductances for repolarizing currents as I_K1_ and I_NaK_ as previously investigated both in human cardiac Purkinje and ventricular models (Passini et al., 2017; Trovato et al., 2020a).

The differences in drug-induced EADs occurrence between *in silico* human and *in vitro* rabbit results are likely to be due to the clear advantage of *in silico* simulations performed in 530 virtual myocytes, rather than a limited number of experiments (4 ≤ *n* ≤ 6 for each drug). In addition, the *in silico* population of models incorporates a large variability in ionic profiles (over- and under-expression of ionic currents), and we previously demonstrated that models with high repolarizing currents and low repolarization reserve are more likely to develop EADs following ion channel blocks, both in ventricular and cardiac Purkinje models (Passini et al., 2017; Trovato et al., 2020b). Therefore, it is much more likely to observe drug induced EADs in human *in silico* drug trials. This is a clear advantage when trying to predict risk, though attention must be paid to possible false positive. In these regards, the accurate assessment of IC50s is crucial for successful adoption of human *in silico* trials in the drug screening pipeline. The coefficients for each ionic conductance of each model in the calibrated population are made available in the Supplement, for reproducibility. The use of a different population of models may lead to slightly different results, though, in previous studies the use of different population of models had no impact on the scientific results (Delaunois et al., 2021).

*In silico* studies such as the one presented here provide valuable information for the classification or ranking of compounds in terms of proarrhythmic risk even early on in drug discovery. Occurrence of EADs in the simulations–even if only observed in few members of the virtual population - are considered as a flag of potential risk warranting further experimentation or investigations into the *in silico* results to identify the underlying ionic profiles of models yielding EADs. Such ionic profiles can be associated with common disease conditions (such as for example low sodium-pump permeability as in ischemic disease) or very rare mutations (resulting for example in low conductance of a particular ionic current). This information is important for decision making and could guide further experiments. No EADs in the simulations are strong digital evidence supporting the safety of the compound, as far as direct effects of the compound to ion channel conductance are concerned. However, this result can be due to the lack of direct effect of the compound, and indirect effects must be ruled out by different experiments, as it is outside of the context of use defined for the *in silico* studies.

Based on the findings of this study, drug safety assessment based on EADs at slow pacing reached perfect accuracy using modelling and simulations of human cardiac Purkinje electrophysiology. In previous studies in ventricular preparations, in addition to EADs-based classification (Passini et al., 2017), high level of accuracy was also reached using the electromechanical window in human ventricular myocytes (Passini et al., 2019). As alternatives, other studies on *in silico* drug safety assessment, consider other tissue models as human stem cell-derived cardiomyocytes (Gintant et al., 2020; Paci et al., 2020), alternative metrics (e.g., qNet (Dutta et al., 2017)), algorithms based on subsets of biomarkers (Champeroux et al., 2005), and approaches combining the population of models (Llopis-Lorente et al., 2022) and/or some degree of machine learning and AI techniques (Varshneya et al., 2021).

We also evaluated how *in silico* and *in vitro* predictions based on drug-induced AP prolongation compare against clinical risk. Although several studies showed that APD increase is not always associated with TdP risk, especially for multichannel blockers (Redfern et al., 2003; Champeroux et al., 2005), it is still one of the most common biomarkers considered in preclinical safety. In our study, predictions based on APD_90_ at 1 Hz reached the same accuracy (86%) *in silico* and *in vitro*, and results were highly consistent (>90%) across all drugs and all concentrations.

Quantitative comparison of drug-induced changes for all biomarkers (dV/dt_MAX_, APD_90_, and APD_50_) and pacing frequencies between human simulations and rabbit preparations also showed large consistency. This is a very exciting result, confirming the importance of developing *in silico* models using experimental data at different frequencies, as we did for the human cardiac Purkinje models used in this study (Trovato et al., 2020a).

Three compounds, i.e., clarithromycin, dofetilide, and sotalol, displayed a larger AP prolongation *in vitro* compared to *in silico*, due to well-known differences between rabbits and humans in responding to hERG block, i.e., larger drug-induced AP prolongation in rabbit. Previous studies have reported smaller clarithromycin-induced AP prolongation in humans compared to rabbit (Gluais et al., 2003), and no QT prolongation at therapeutic doses (Démolis et al., 2003). Also, several studies have reported in rabbit the largest dofetilide-induced AP prolongation, compared to other species e.g. humans, dog, guinea pig, swine, goat, sheep (Lu et al., 2001; Lu et al., 2002; Terrar et al., 2007; Trovato et al., 2020b). Previous studies also showed larger sotalol-induced AP prolongation in rabbit compared to other species (Gintant et al., 2001), and AP prolongation between 28% and 37% following superinfusion of 30 µM sotalol in human cardiomyocytes (Tveito et al., 2020), closer to what was observed in our simulations (16%) than in rabbit experiments (96%).

To our knowledge, this is the first study that systematically evaluates and compares homogenous experimental data capturing drug-induced effect on cardiac Purkinje fibers against *in silico* results. To minimize noise and variability in the experimental electrophysiological recordings, we considered a consistent dataset, with experiments performed in one laboratory and under identical conditions. IC50s values, instead, were considered from multiple sources, introducing additional noise, which could represent a limitation of this study, although the accuracy in the classification was very high.

In summary, the credibility goals that we satisfied in this study, as defined in (Musuamba et al., 2021), are: 1) to show higher accuracy of *in silico* trials compared to a current-in-use experimental counterpart; 2) to demonstrate high grade of consistency between simulations and experiments. Our results showed not only high degree of consistency between *in vitro* and *in silico* preparations (Figure 6), but also that human-based computer models and simulations can achieve better results than rabbit experiments for risk predictions (Figure 4A) since they are built, calibrated, and validated using human data, thus facilitating translation towards clinical scenarios.

In addition, there are more advantages in using *in silico* model compared to perform *in vitro* animal experiments. These include: 1) reduction of the use of animals in research; 2) reduction of the time required for drug safety assessment, thus allowing pharmaceutical companies to process more compounds in a shorter amount of time, and accelerate the drug development process; 3) economical advantage, i.e., *in silico* trials can be performed in a standard computer; 4) overcome limitations in the tested concentration ranges, due to drug solubility problems; 5) overcome limitations in the number of conditions explored for each drug (concentrations, pacing frequencies, etc.) and in the number of samples.

To conclude, *in silico* drug trials in human cardiac Purkinje cells have shown to be consistent with *in vitro* recordings from rabbit Purkinje fibers, and more accurate for predictions of drug-induced proarrhythmic risk. This study contributes evidence towards the vision of *in silico* trials for preclinical drug assessment as alternative to animal models.

## Data Availability

The original contributions presented in the study are included in the article/Supplementary Material, further inquiries can be directed to the corresponding author.
